# Sex-dependent regulation of retinal pigment epithelium and retinal function by *Pgc-1α*

**DOI:** 10.3389/fncel.2024.1442079

**Published:** 2024-09-02

**Authors:** Kaan Taskintuna, Mohd Akbar Bhat, Tasneem Shaikh, Jacob Hum, Nady Golestaneh

**Affiliations:** ^1^Department of Ophthalmology, Georgetown University Medical Center, Washington, DC, United States; ^2^Department of Pharmacology and Physiology, Georgetown University Medical Center, Washington, DC, United States; ^3^Department of Biochemistry and Molecular and Cellular Biology, Georgetown University Medical Center, Washington, DC, United States; ^4^Department of Neurology, Georgetown University Medical Center, Washington, DC, United States

**Keywords:** RPE, retina, PGC-1α, mitochondria, AMD, aging, sex-difference

## Abstract

Age-related macular degeneration (AMD) is a major cause of blindness that affects people over 60. While aging is the prominent factor in AMD, studies have reported a higher prevalence of AMD in women compared to age-matched men. Higher levels of the innate immune response’s effector proteins complement factor B and factor I were also found in females compared to males in intermediate AMD. However, the mechanisms underlying these differences remain elusive. Peroxisome proliferator-activated receptor gamma coactivator-1 alpha (PGC-1α) is a key regulator of mitochondrial biogenesis and metabolic pathways. Previously, we showed that *Pgc-1α* repression and high-fat diet induce drastic AMD-like phenotypes in mice. Our recent data revealed that *Pgc-1α* repression alone can also induce retinal pigment epithelium (RPE) and retinal dysfunction in mice, and its inhibition *in vitro* results in lipid droplet accumulation in human RPE. Whether sex is a contributing factor in these phenotypes remains to be elucidated. Using electroretinography, we demonstrate that sex could influence RPE function during aging independent of *Pgc-1α* in wild-type (WT) mice. We further show that *Pgc-1α* repression exacerbates RPE and retinal dysfunction in females compared to aged-match male mice. Gene expression analyses revealed that *Pgc-1α* differentially regulates genes related to antioxidant enzymes and mitochondrial dynamics in males and females. RPE flat mounts immunolabeled with TOMM20 and DRP1 indicated a sex-dependent role for *Pgc-1α* in regulating mitochondrial fission. Analyses of mitochondrial network morphology suggested sex-dependent effects of *Pgc-1α* repression on mitochondrial dynamics. Together, our study demonstrates that inhibition of *Pgc-1α* induces a sex-dependent decline in RPE and retinal function in mice. These observations on the sex-dependent regulation of RPE and retinal function could offer novel insights into targeted therapeutic approaches for age-related RPE and retinal degeneration.

## Introduction

Age-related macular degeneration (AMD) is a major cause of irreversible vision loss among the elderly. Its pathophysiology is characterized by degeneration of the retinal pigment epithelium (RPE) and the ensuing loss of photoreceptors, leading to central vision impairment (O’Shea, 1996). AMD is a multifactorial disease with genetic, environmental, dietary, and metabolic factors (Gehrs et al., 2006; Bok, 1993; Boulton and Dayhaw-Barker, 2001; Bowes Rickman et al., 2013), where aging remains the primary risk factor (Zhang et al., 2008). There are sex-dependent differences in how the visual system ages. Males and females demonstrate differences in changes to retinal disparity thresholds (Zaroff et al., 2003). Race and ethnicity have also been related to the prevalence of AMD (Butt et al., 2011; McGowan et al., 2014; Frank et al., 2000). A higher prevalence of AMD in women compared to age-matched men were identified in a Japanese population (Sasaki et al., 2018). Higher levels of complement factor B, responsible for the amplification of the innate immune response (Brown et al., 2023) and factor I, an essential negative regulator of complement activation (de Jong et al., 2021) are found in females compared to males with intermediate AMD (Marin et al., 2022). Additionally, a potentially higher risk of neovascular AMD for women has been reported (Rudnicka et al., 2012). Another study has found an association between a higher risk of developing early AMD in women with polymorphisms in AMD risk genes (Sasaki et al., 2018). Despite the data suggesting a higher prevalence of AMD in females, the mechanisms underlying the sex-dependent differences in AMD remain largely unknown. Mitochondrial dysfunction has been associated with the pathophysiology of AMD (Golestaneh et al., 2016; Ferrington et al., 2017; Kaarniranta et al., 2020). Dysfunctional mitochondria induce increased levels of reactive oxygen species (ROS) and defective metabolic activity (Jarrett et al., 2013). Mitochondria are suggested to be a “central target for sex differences in diseases” (Ventura-Clapier et al., 2017). Evidence from a wide variety of pathologies, ranging from central nervous system disorders to cardiomyopathies, suggests fundamental differences in the involvement of biological sex in mitochondrial dysfunction, impacting disease prevalence, progression, and response to treatment (Ventura-Clapier et al., 2017; Demarest and McCarthy, 2015).

It remains to be elucidated whether mitochondrial function is affected in a sex-dependent manner in the RPE and retina during normal aging and in AMD. Peroxisome proliferator-activated receptor gamma coactivator-1 alpha (PGC-1α; encoded by the gene *PPARGC1A*) is a transcriptional coactivator that plays a central role in mitochondrial biogenesis and oxidative metabolism (Liang and Ward, 2006). PGC-1α is highly expressed in metabolically active tissues such as the RPE/retina (Egger et al., 2012), brown adipose tissue, skeletal muscle, kidneys, and heart (Cheng et al., 2018). Beyond mitochondrial biogenesis and oxidative metabolism, studies have shown far-reaching regulatory functions of PGC-1α in crucial homeostatic processes, including lipid metabolism, mitochondrial dynamics (Martin et al., 2014), mitochondrial quality control (Qian et al., 2024), antioxidant defense (Rius-Perez et al., 2020), and autophagy (Vainshtein et al., 2015).

In the RPE and retina, PGC-1α has been shown to protect the aging RPE from oxidative stress and activate oxidative phosphorylation (Kaarniranta et al., 2018; Gurubaran et al., 2024; Iacovelli et al., 2016). Its repression is associated with RPE epithelial-to-mesenchymal transition (Rosales et al., 2019), induction of pathological retinal angiogenesis (Saint-Geniez et al., 2013), and light-induced apoptosis in the retina (Egger et al., 2012).

Previously, we reported increased acetylation of PGC-1α (resulting in its inactivation) and decreased activity of related energy-sensing pathways in RPE derived from AMD donor eyes (Zhang et al., 2020). Our recent work showed that inhibition of *PGC1A* in human RPE induces lipid droplet accumulation (Zhou et al., 2024). Our past research also showed that heterozygous repression of *Pgc-1α* in mice (*Pgc-1α*^+/−^), combined with a high-fat diet, induces AMD-like degenerative phenotypes (Zhang et al., 2018). These observations suggest that the *Pgc-1α*^+/−^ mice are a valuable animal model to study the RPE/retinal functions and metabolism *in vivo*. Our established *Pgc-1α*^+/−^ mouse model is physiologically more relevant to humans as it reflects a decline instead of a complete loss of gene expression. In addition, studies have shown that null *Pgc-1α* models result in a compensatory increase in *Pgc-1β* (Besse-Patin et al., 2017).

A complex interplay between the sex hormone estradiol and PGC-1α in the liver has been reported. In addition, loss of estrogen signaling in females was associated with increased susceptibility to oxidative stress (Besse-Patin et al., 2017; Herrera-Marcos et al., 2020).

Here, we aim to investigate the impact of biological sex on RPE and retinal health in the context of aging and *Pgc-1α* repression. Understanding the sex-dependent mechanisms by which *Pgc-1α* regulates RPE and retinal function could guide the development of targeted therapies for RPE and retinal degeneration.

## Materials and methods

### Animals

Studies involving animals were approved by the Institutional Animal Care and Use Committee of Georgetown University and were in compliance with the Statement for the Use of Animals in Ophthalmic and Vision Research from the Association for Research in Vision and Ophthalmology (ARVO). Heterozygous B6.129S4(FVB)-*Ppargc1a^tm1Brsp^*/J mice on a C57BL/6J background were originally purchased from the Jackson Laboratory (Bar Harbor, ME, United States) and bred to generate whole-body heterozygous (*Pgc-1α*^+/−^) mice as well as wild-type (WT) littermates. Animals were kept in a temperature- and humidity-controlled room on a 12-h dark:12-h light cycle with access to water and food *ad libitum*. Automated genotyping from tail biopsies was performed by TransnetYX (Cordova, TN, United States). Three months-old (3mo) and eight months-old (8mo) WT and *Pgc-1α*^+/−^ animals of both sexes were used in electroretinogram (ERG) procedures. Animals were euthanized with standard CO_2_ euthanasia and subsequent confirmatory cervical dislocation, according to Georgetown University Institutional Animal Care and Use Committee and American Veterinary Medical Association guidelines.

### qRT-PCR

Total RNA was extracted from RPE/retina of 8mo WT and *Pgc-1α*^+/−^ animals of both sexes using the Omega BioTek E.Z.N.A. Total RNA Kit (R6834; Omega BioTek, Norcross, GA, United States). Reverse transcription of RNA and SYBR-Green based quantitative real-time PCR (qPCR) on a Bio-Rad CFX Connect system (Bio-Rad, Hercules, CA, United States) were performed as previously described (Zhou et al., 2023). *Gapdh* was used as the reference gene, and fold changes were calculated normalizing to a randomly selected WT male mouse using the 2^−ΔΔCT^ method qPCR ANOVA tables. Mouse gene primer sequences, located on separate exons, were designed using Primer BLAST and PrimerBank. Primers were ordered from Integrated DNA Technologies (IDT, Coralville, Iowa, United States). Forward and reverse primer sequences for corresponding genes are listed in Supplementary Table S1.

### Electroretinogram

Mice were dark-adapted overnight. Induction of anesthesia was carried out with 3.0% isoflurane and 0.8 L/min oxygen in an induction chamber under dim red-light conditions for 3 min. Then, they were transferred onto the heated surface of the Diagnosys Celeris (Diagnosys LLC, Lowell, MA, United States) platform, kept at a constant 37°C where 1.5% isoflurane with 0.8 L/min oxygen was continuously delivered through a nose cone for maintenance. 2.5% Phenylephrine and 1% Tropicamide eye drops were used to induce mydriasis. The ERG protocols were carried out as previously described (Zhou et al., 2024). We used the dark-adapted flash ERG to measure: (i) a-waves representing rod photoreceptor responses; (ii) b-waves, representing responses primarily generated by ON-bipolar cells and Müller glia (Asanad and Karanjia, 2024). c-wave ERG was performed to assess RPE function (Perlman, 1995). Amplitudes and implicit times (time elapsed from stimulus onset to peak recorded amplitude) from right and left eyes were averaged into one data point for each animal.

### RPE flat mounts

RPE flat mounts from 8-month-old mice were performed by fixing the posterior eyecups for 40 min in 4% paraformaldehyde. For 30 min at room temperature, the eyecups were placed on an orbital shaker in a blocking solution containing: 1% bovine serum albumin (BSA), 5% normal goat serum, and 0.3% Triton-X in PBS. To block endogenous mouse immunoglobulins, 1 h of blocking was performed with the Mouse-on-Mouse Blocking Reagent (BMK-2202; Vector Laboratories, Newark, CA, United States). Samples were incubated with primary antibodies for 30 min at room temperature, followed by 4°C overnight incubation in an antibody solution containing 1% BSA, 1% normal goat serum, and 0.1% Triton-X in PBS. Secondary antibodies were incubated for 1.5 h at room temperature, along with counterstains DAPI for nuclei (1:1000 dilution; sc-3598; Santa Cruz Biotechnology, Santa Cruz, CA, United States), Texas Red-X Phalloidin for cytoskeletal actin filaments (1:800 dilution; T7471; Life Technologies, Carlsbad, CA, United States). A drop of VectaShield Plus Antifade Mounting Medium (H-1900; Vector Laboratories) was placed on a 22 × 22 coverslip and gently placed on top of the tissue.

Mitochondrial staining was performed with mouse anti-TOMM20 antibody (1:150 dilution; ab56783; Abcam; Boston, MA, United States). Rabbit anti-DRP1 (1:100 dilution; 8570; Cell Signaling Technology, Danvers, MA, United States). Secondary antibodies used were AlexaFluor 488 goat anti-mouse (1:500 dilution; A11001; Invitrogen; Waltham, MA, United States) and AlexaFluor 647 goat anti-rabbit (1:500 dilution; A32733; Invitrogen).

### Image processing and analysis

Confocal microscopy of RPE flat mounts was performed using the Leica TCS SP8 AOBS laser scanning confocal microscope using a 63× immersion oil objective and 3× digital zoom. 2D Images of RPE flatmounts were deconvoluted using the Huygens Professional Software (SVI, Hulversum, Netherlands). Colocalization analyses of immunolabeled DRP1 and TOMM20 were performed on Huygens Professional on selected regions of interest (ROI) encompassed whole cells within the field of view. The output value for statistical comparisons of colocalization was Pearson’s Correlation coefficient (Adler and Parmryd, 2010).

Mitochondrial network analysis was performed using the Mitochondria Analyzer plugin on Fiji (Chaudhry et al., 2020). The settings used for thresholding were determined according to established 2D analysis methods by the developers of Mitochondria Analyzer (Chaudhry et al., 2020). Settings used are as follows: “Local Threshold Method: Weighted Mean”; “Gamma: 0.80”; “Max Slope: 1.80”; “Sigma Filter Plus Radius: 1.70”; “Subtract Background: 1.25”; “C-value: 3”; “Block Size: 1.55 microns”; “Outlier Radius: 2.4 pixels.” Thresholded binary images obtained through Mitochondria Analyzer were skeletonized and analyzed using the “Analyze Skeleton 2D” function.

### Statistical analysis

For comparison of three independent variables on one dependent variable for c-wave ERG, (factors: age × sex × genotype) three-way ANOVA with *post hoc* Holm-Šídák test was performed. The factor age is 3mo vs. 8mo, sex is male vs. female, and genotype is WT vs. *Pgc-1α*^+/−^. For dark-adapted flash ERG a- and b-waves, the data were analyzed via two-way repeated measures ANOVA with *post hoc* Holm-Šídák test for multiple comparisons to identify main effects of sex/genotype × flash intensity on ERG amplitudes across a series of ascending flash intensities.

Gene expression and imaging analyses were performed on data obtained from 8mo animals. Two-way ANOVA with *post hoc* Holm-Šídák test for multiple comparisons (factors: sex × genotype) was performed.

ANOVA tables for gene qPCR data can be found in Supplementary Tables S4, S5. ANOVA tables for imaging analyses can be found in Supplementary Tables S6, S7. Logarithmic transformation of data was carried out to meet assumptions of ANOVA homoscedasticity and normality of residuals when appropriate. Log-transformed values are indicated as such on the *y*-axes of corresponding graphs. Data are presented as mean ± SEM. Statistical analyses and graphing of data were performed using GraphPad Prism 10.2.3.

## Results

### Sex- and age-dependent decline of RPE function in WT and *Pgc-1α*^+/−^ mice

**Figure 1:**
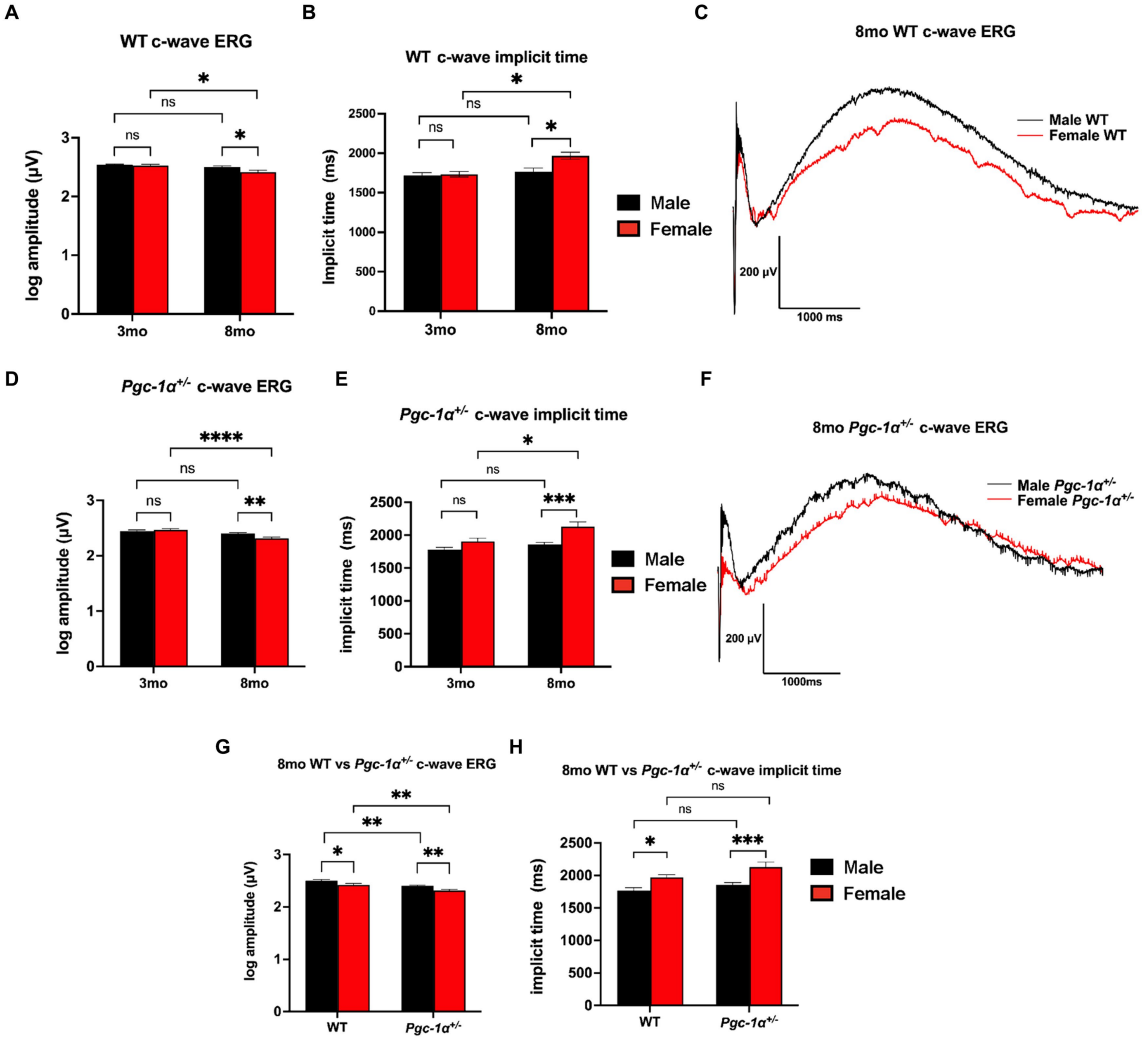
Sex-dependent regulation of RPE function. **(A)** c-wave ERG amplitudes of 3mo vs. 8mo male and female WT mice. **(B)** c-wave ERG implicit times (duration between light stimulus onset and peak amplitude) of 3mo vs. 8mo male and female WT mice. **(C)** Representative c-wave ERG traces of 8mo male vs. female WT mice. **(D)** c-wave ERG amplitudes of 3mo vs. 8mo male and female *Pgc-1α*^+/−^ mice. **(E)** c-wave ERG implicit times of 3mo vs. 8mo male and female *Pgc-1α*^+/−^ mice. **(F)** Representative c-wave ERG traces of 8mo male vs. female *Pgc-1α*^+/−^ mice. **(G)** c-wave ERG amplitudes of 8mo WT and *Pgc-1α*^+/−^ male and female mice. **(H)** c-wave ERG implicit times of 8mo WT and *Pgc-1α*^+/−^ male and female mice. Data presented are representations of *post hoc* Holm-Šídák test for multiple comparisons of three-way ANOVA (factors: age, sex, genotype). *n* = 13–18 mice per group; exact sample size for each group is listed in Supplementary Table S2. Mean ± SEM. ^*^p ≤ 0.05, ^**^p ≤ 0.01, ^***^p ≤ 0.001, and ^****^p ≤ 0.0001.

We performed c-wave ERG to evaluate RPE function in 3mo and 8mo WT and *Pgc-1α*^+/−^ mice. Comparison of c-wave ERG amplitudes between 3mo and 8mo male and female WT mice showed a statistically significant decline with aging in WT females (adjusted *p* = 0.0141), but not in WT males (Figure 1A). 8mo WT females also showed significantly lower c-wave amplitudes compared to 8mo WT males (Figures 1A, C; adj. *p* = 0.0368). No sex differences were observed at the younger age of 3mo between male and female WT (Figure 1A). c-wave implicit times (time elapsed between flash stimulus onset to peak amplitude) for 8mo WT females were significantly delayed when compared to 3mo WT females (Figure 1B; adj. *p* = 0.0284). For WT males, there were no statistically significant differences in c-wave implicit times when comparing 3mo and 8mo male WT (Figure 1B). Additionally, significantly delayed c-wave implicit times were observed in 8mo WT females compared to 8mo WT males (adj. *p* = 0.0441). There were no sex differences at 3mo when comparing male and female WT mice (Figure 1B).

For *Pgc-1α*^+/−^ mice, c-wave amplitudes showed a statistically significant decline with aging in females (adj. *p* < 0.0001), but not in males (Figure 1D). Furthermore, c-wave amplitudes were found to be significantly lower in female 8mo *Pgc-1α*^+/−^ compared to males (Figures 1D, F; adj. *p* = 0.0092). 8mo *Pgc-1α*^+/−^ females had significantly higher implicit times compared to 8mo males (Figure 1E; adj. *p* = 0.0006). Comparison of 3mo *Pgc-1α*^+/−^ and 8mo *Pgc-1α*^+/−^ females revealed significantly delayed implicit times in 8mo females (Figure 1E; *p* = 0.0148). In male *Pgc-1α*^+/−^ mice, age did not have a statistically significant effect on c-wave implicit times. There were no sex differences at 3mo when comparing male and female *Pgc-1α*^+/−^ mice c-wave implicit times.

We observed no significant differences in c-wave amplitudes for WT and *Pgc-1α*^+/−^ mice at the age of 3mo within each sex (Supplementary Table S1). At the age of 8mo, both male and female *Pgc-1α*^+/−^ mice showed significantly lower c-wave amplitudes compared to their WT counterparts (Figure 1G; adj. *p* = 0.0092, 0.0033, respectively). *Pgc-1α* repression significantly decreased c-wave ERG amplitudes irrespective of sex compared to WT, but did not significantly affect implicit times (Figures 1G, H).

Three-way ANOVA analysis (factors: age × sex × genotype) revealed a significant two-way interaction effect of age × sex on c-wave amplitudes (*p* = 0.0044). The main effects of sex (*p* = 0.0014), age (*p* < 0.0001), and genotype (*p* < 0.0001), on c-wave amplitudes were all found to be significant. Three-way ANOVA analysis of c-wave implicit times also revealed a significant two-way interaction effect between age × sex (*p* = 0.0340) as well as significant main effects for age (*p* < 0.0001), sex (*p* < 0.0001), and genotype (*p* = 0.0016).

### Sex-dependent decline of photoreceptor function in *Pgc-1α*^+/−^ mice

**Figure 2:**
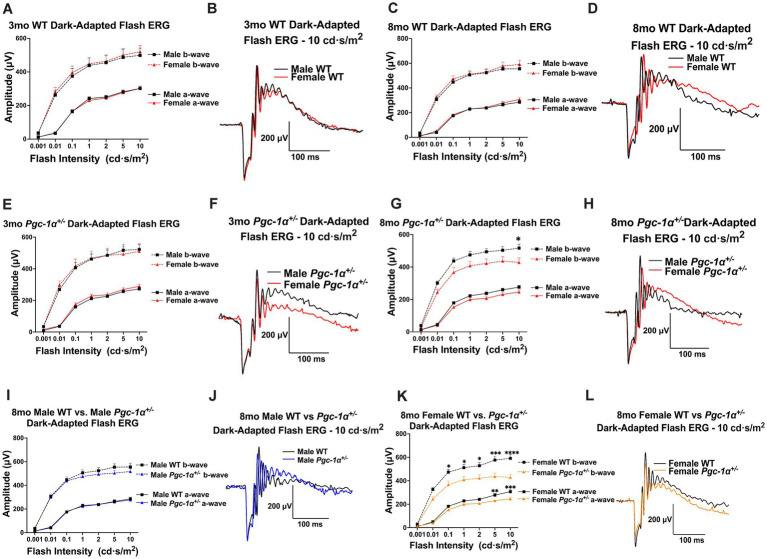
Sex-dependent effects of *Pgc-1α* repression on photoreceptor and inner retinal function. **(A)** Dark-adapted flash ERG a- and b-wave amplitudes in 3mo male vs. female WT mice across seven stimulus intensities ranging from 0.001–10.0 cd·s/m^2^. **(B)** Representative dark-adapted flash ERG traces of 3mo male and female WT mice at 10.0 cd·s/m^2^. **(C)** a- and b-wave amplitudes of 8mo male and female WT mice. **(D)** Representative dark-adapted flash ERG traces of 8mo male and female WT mice. **(E)** a- and b-wave amplitudes of 3mo male and female *Pgc-1α*^+/−^ mice. **(F)** Representative dark-adapted flash ERG traces of 3mo male and female *Pgc-1α*^+/−^ mice. **(G)** a- and b-wave amplitudes of 8mo male and female *Pgc-1α*^+/−^ mice. **(H)** Representative dark-adapted flash ERG traces of 8mo male and female *Pgc-1α*^+/−^ mice. **(I)** a- and b-wave amplitudes of 8mo male WT and 8mo *Pgc-1α*^+/−^ mice. **(J)** Representative dark-adapted flash ERG traces of 8mo male WT and male *Pgc-1α*^+/−^ mice. **(K)** a- and b-wave amplitudes of 8mo female WT and female *Pgc-1α*^+/−^ mice. **(L)** Representative dark-adapted flash ERG traces of 8mo female WT and female *Pgc-1α*^+/−^. *n* = 12–20 mice per group; precise sample size for each group is listed in Supplementary Table S3. Two-way repeated measures ANOVA with *post hoc* Holm-Šídák test for multiple comparisons. Mean ± SEM. ^*^p ≤ 0.05, ^**^p ≤ 0.01, ^***^p ≤ 0.001, and ^****^p ≤ 0.0001.

Dark-adapted flash ERG was used to assess rod photoreceptor and inner retinal function in WT and *Pgc-1α*^+/−^ mice. At the age of 3mo, we did not observe statistically significant effects of sex in a- or b-wave amplitudes in WT mice (Figure 2A, B). 8mo WT males and females did not show any statistically significant differences in a- or b-wave amplitudes (Figure 2C, D).

There were no statistically significant sex differences in a- or b-wave amplitudes in 3mo *Pgc-1α*^+/−^ males and females (Figure 2E, F). However, for 8mo *Pgc-1α*^+/−^ males and females, we observed a significant main effect of sex on b-wave amplitudes (*p* = 0.0376), but not for a-wave amplitudes (Figure 2G). *Post hoc* analyses revealed significantly higher average b-wave amplitudes for 8mo *Pgc-1α*^+/−^ males compared to females at the maximum flash intensity of 10.0 cd·s/m^2^ (Figure 2F, G; adj. *p* = 0.0461).

We did not observe statistically significant differences between 8mo WT males and 8mo *Pgc-1α*^+/−^ males in a- or b-wave responses (Figure 2I, J). Between 8mo WT females and 8mo *Pgc-1α*^+/−^ females, we observed a significant effect of genotype on a-waves (*p* = 0.0184), as well as b-waves (*p* = 0.0041) (Figure 2K). Significantly lower a-wave amplitudes in 8mo *Pgc-1α*^+/−^ females were found at the flash intensity values of 5.0 cd·s/m^2^ (adj. *p* = 0.0068) and 10.0 cd·s/m^2^ (adj. *p* = 0.0002). For b-waves, we observed significantly lower b-wave amplitudes in *Pgc-1α*^+/−^ females compared to WT females at the flash intensities of 0.01, 0.1, 1.0, 2.0, 5.0, and 10.0 cd·s/m^2^ (Figure 2K, L; adj. *p* = 0.0491; 0.0184; 0.0184; 0.0184; 0.0009; <0.0001; respectively). Significant differences were not observed in a- or b-wave implicit times across any comparisons (data not shown).

We investigated the effects of sex or genotype on ERG a- and b-wave amplitudes using repeated measures two-way ANOVA (factors: sex × flash intensity or genotype × flash intensity). We carried out analyses of (i) sex differences within each genotype at each age; (ii) the effect of genotype within sexes at the same age.

### Gene expression differences in antioxidant enzymes

PGC-1*α* plays an important role in mitochondrial biogenesis, lipid metabolism, and the regulation of encoding genes for antioxidant enzymes (Cheng et al., 2018).

We performed gene expression analyses of antioxidant enzymes and transcription factors involved in the regulation of reactive oxygen species (ROS) scavenging pathways.

**Figure 3:**
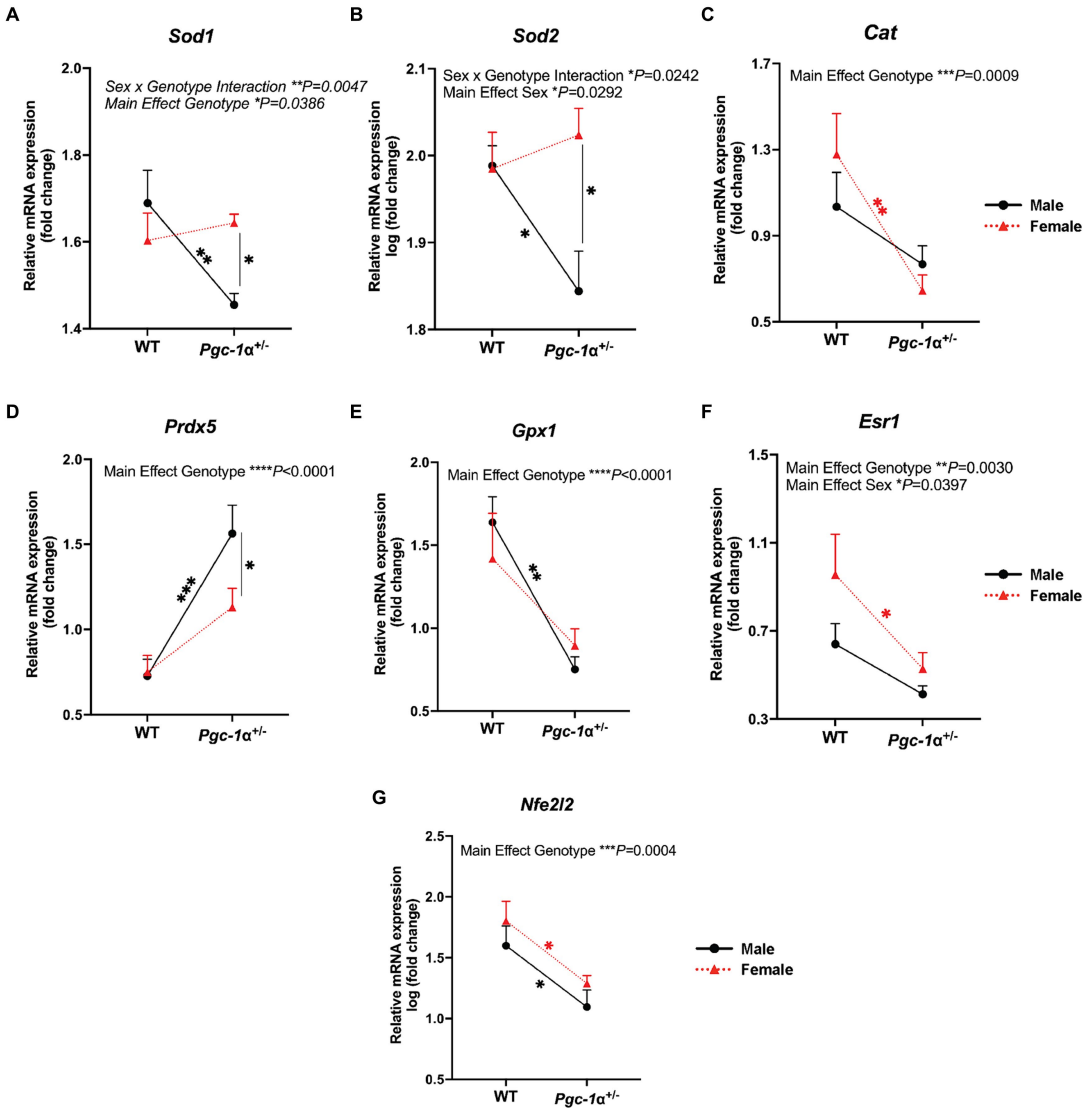
The effect of sex and *Pgc-1α* repression on the transcriptional regulation of antioxidant enzymes. Comparison of the relative gene expression of antioxidant enzymes and upstream transcriptional regulators in 8mo male and female WT and *Pgc-1α*^+/−^ mice, represented by interaction plots. **(A)** *Sod1* (superoxide dismutase 1). **(B)** *Sod2* (superoxide dismutase 2). **(C)** *Cat* (catalase). **(D)** *Prdx5* (peroxiredoxin 5). **(E)** *Gpx1* (gluthathione peroxidase 1). **(F)** *Esr1* (estrogen receptor alpha). **(G)** *Nfe2l2* (nuclear factor erythroid 2-related factor 2). *n* = 6–11 animals per group. Four pairwise comparisons made for each gene consisted of within-sex and within-genotype comparisons. Two-way ANOVA with *post hoc* Holm-Šídák test for multiple comparisons. Mean ± SEM. ^*^p ≤ 0.05, ^**^p ≤ 0.01, ^***^p ≤ 0.001, and ^****^p ≤ 0.0001.

For the cytosolic form of superoxide dismutase (Mondola et al., 2016), *Sod1* (copper-zinc superoxide dismutase), we found a significant interaction effect of sex and genotype (*p* = 0.0047) and main effect of genotype (*p* = 0.0386). *Post hoc* analyses revealed a significant decrease in *Sod1* mRNA levels in male *Pgc-1α*^+/−^ mice compared to WT males (adj. *p* = 0.0047) and compared to *Pgc-1α*^+/−^ females (Figure 3A; adj. *p* = 0.0075). mRNA levels of the mitochondrial form of superoxide dismutase (Liu et al., 2022), *Sod2* (manganese superoxide dismutase), showed a significant interaction effect between sex and genotype (*p* = 0.0242) and a significant main effect of sex (Figure 3B; *p* = 0.0292). The expression of *Sod2* was found to be significantly decreased in male *Pgc-1α*^+/−^ compared to male WT (adj. *p* = 0.0437) and *Pgc-1α*^+/−^ female mice (Figure 3B) (adj. *p* = 0.0040).

Catalase (*Cat*), an essential antioxidant enzyme responsible for the breakdown of hydrogen peroxide (Nandi et al., 2019), is crucial to RPE antioxidant defense mechanisms (Kaarniranta et al., 2018). We observed a significant main effect of genotype on relative *Cat* mRNA levels (*p* = 0.0009). The expression of *Cat* was found to be significantly downregulated in *Pgc-1α*^+/−^ females compared to WT females (Figure 3C; adj. *p* = 0.0032).

Peroxiredoxin 5 (PRDX5) is an antioxidant enzyme that uses thioredoxins to reduce alkyl hydroperoxides and hydrogen peroxide (Perkins et al., 2015). mRNA levels of *Prdx5* was significantly upregulated in Pgc. We observed a significant upregulation of *Prdx5* in male *Pgc-1α*^+/−^ mice compared to WT males (adj. *p* = 0.0007). *Prdx5* expression was not significantly upregulated in *Pgc-1α*^+/−^ females compared to WT females. Moreover, *Prdx5* expression in male *Pgc-1α*^+/−^ was significantly higher compared to *Pgc-1α*^+/−^ females (Figure 3D; adj. *p* = 0.046).

Gluthathione peroxidase 1, *Gpx1*, involved in the catalyzing the reduction of reduced gluthathione and hydrogen peroxide (Handy and Loscalzo, 2022), showed a main effect of genotype (Figure 3E; *p* < 0.0001). In both *Pgc-1α*^+/−^ males and females, *Gpx1* mRNA levels were significantly reduced compared to WT mice irrespective of sex (adj. *p* = 0.0003; 0.0075, respectively).

Estrogen receptor alpha, encoded by the gene *Esr1*, has been reported to signal through *Pgc-1α* to mediate antioxidant responses against oxidative stress (Besse-Patin et al., 2017). We found significant main effects of sex and genotype on *Esr1* mRNA levels (Figure 3F; *p* = 0.0030; *p* = 0.0397, respectively). Pairwise comparisons showed a significant decrease in *Esr1* gene expression in *Pgc-1α*^+/−^ females compared to WT females (adj. *p* = 0.0314).

*Nfe2l2* (also known as *Nrf2*), is a well-established transcriptional partner of PGC-1α (Gureev et al., 2019). NFE2L2 is a master regulator of antioxidant defense and sensor of oxidative stress (Gureev et al., 2019). We observed a strong decline in *Nfe2l2* expression in *Pgc-1α*^+/−^ mice, irrespective of sex (Figure 3G; main effect genotype: *p* = 0.0004). Statistical analyses were performed using two-way ANOVA (factors: genotype × sex) on gene expression data from RPE/retina extracts of 8mo WT and *Pgc-1α*^+/−^ mice of both sexes.

### Gene expression differences in key mediators of mitochondrial dynamics

Next, we investigated whether *Pgc-1α* repression exerts sex-dependent effects on the mRNA levels of genes involved in mitochondrial fission, fusion, and biogenesis.

**Figure 4:**
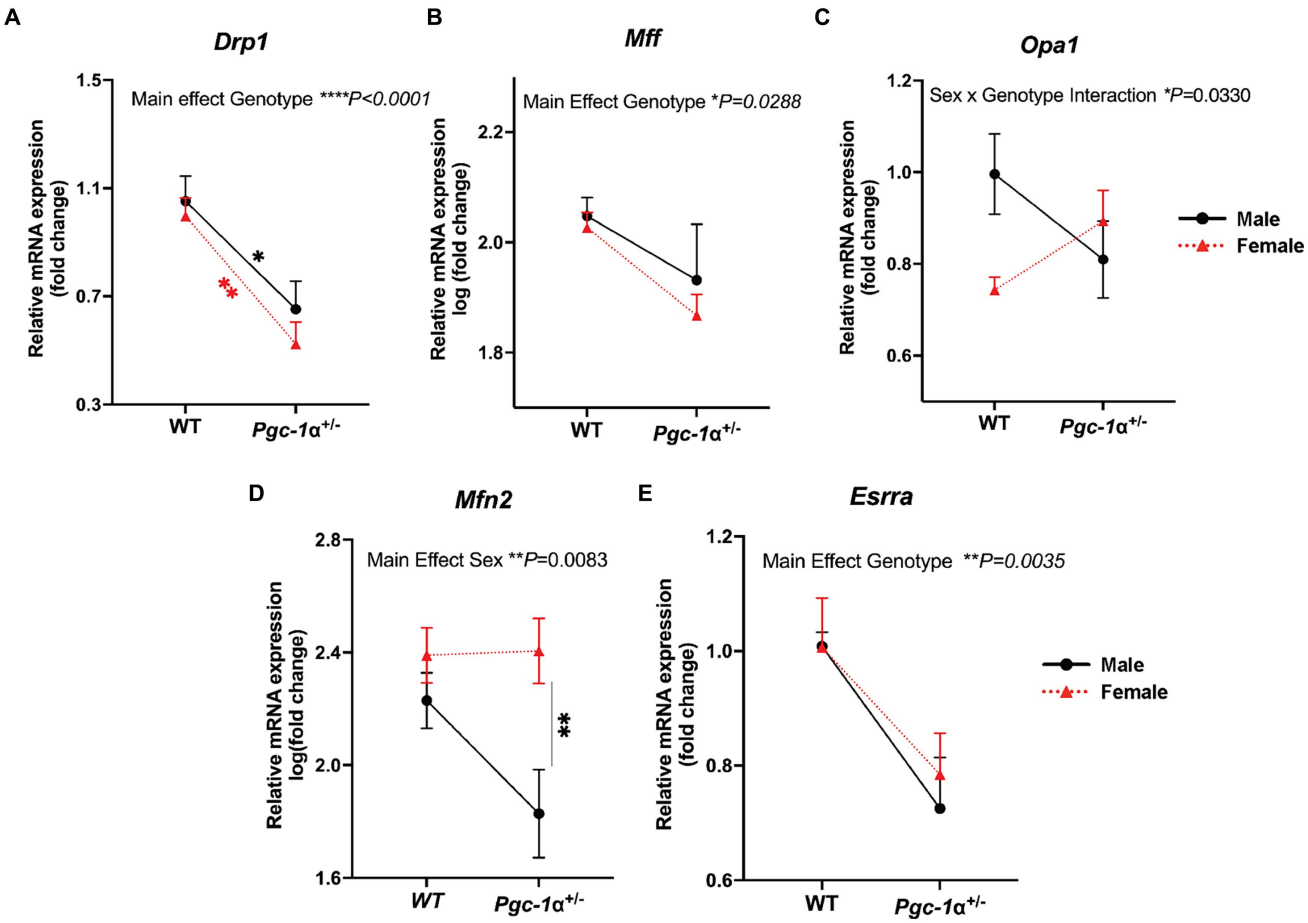
Differential regulation of genes related to mitochondrial dynamics in *Pgc-1α*^+/−^ males and females. Comparison of the relative gene expression of mitochondrial dynamics proteins in 8mo male and female WT and *Pgc-1α*^+/−^ mice, represented by interaction plots. **(A)** *Drp1* (dynamin-1-like protein). **(B)** *Mff* (mitochondrial fission factor). **(C)** *Opa1* (optic atrophy 1). **(D)** *Mfn2* (mitofusin 2). **(E)** *Esrra* (estrogen-related receptor alpha). Four pairwise comparisons made for each gene consisted of within-sex and within-genotype comparisons. *n* = 6–11 animals per group. Two-way ANOVA with *post hoc* Holm-Šídák test for multiple comparisons. Mean ± SEM. ^*^p ≤ 0.05, ^**^p ≤ 0.01, ^***^p ≤ 0.001, and ^****^p ≤ 0.0001.

Dynamin-related protein 1 (DRP1) is a GTPase required for the initiation of mitochondrial fission (Kleele et al., 2021). Through its recruitment to the mitochondrial outer membrane and interaction with adapter proteins, the oligomerization of DRP1 allows constriction at designated mitochondrial sites to facilitate mitochondrial fission (Kleele et al., 2021). We found a significant main effect of *Pgc-1α* repression (*p* < 0.0001) on reducing *Drp1* expression compared to WT for both sexes (Figure 4A; adj. *p* = 0.0164 for male WT vs. male *Pgc-1α*^+/−^; adj. *p* = 0.0044 for female WT vs. female *Pgc-1α*^+/−^).

Mitochondrial fission factor, MFF, is an adaptor protein for DRP1 oligomers on the outer mitochondrial membrane that facilitates midline division of mitochondria for mitochondrial biogenesis (Kleele et al., 2021). A significant main effect of *Pgc-1α* repression on reducing *Mff* mRNA levels was found (*p* = 0.0288), however, *post hoc* analyses were unable to detect significant pairwise differences (Figure 4B).

*Opa1*, a dynamin-related GTPase, is an essential protein for the fusion of the inner mitochondrial membrane as well as the maintenance of mitochondrial cristae (Ge et al., 2020). The complex posttranslational regulation of OPA1 is highly nuanced and it can also play a role in mitochondrial fission (Ge et al., 2020). Here, we found a significant interaction effect of Genotype and Sex on *Opa1* expression (*p* = 0.0330) (Figure 4C).

Mitofusin 2, *Mfn2*, is an outer mitochondrial GTPase critical for mitochondrial fusion (Adebayo et al., 2021). We observed a significant main effect of sex (Figure 4D; *p* = 0.0083). Pairwise comparisons revealed a significantly higher expression of *Mfn2* in *Pgc-1α*^+/−^ females compared to *Pgc-1α*^+/−^ males (adj. *p* = 0.0061).

Estrogen-related receptor alpha, *Esrra*, is a transcriptional partner of *Pgc-1α* in the regulation of mitochondrial biogenesis and many aspects of oxidative metabolism (Zhu et al., 2010). *Esrra*, through common pathways with *Pgc-1α* as well as through distinct mechanisms, has been reported to regulate DRP1-mediated mitochondrial fission both within the context of mitochondrial biogenesis and mitophagy (Tripathi et al., 2020). Our analysis revealed a significant main effect of genotype (Figure 4E; *p* = 0.0035) on *Esrra* expression. Two-way ANOVA (factors: genotype × sex) was used for statistical analyses.

### Sex differences in the regulation of RPE mitochondrial dynamics by *Pgc-1α*

**Figure 5:**
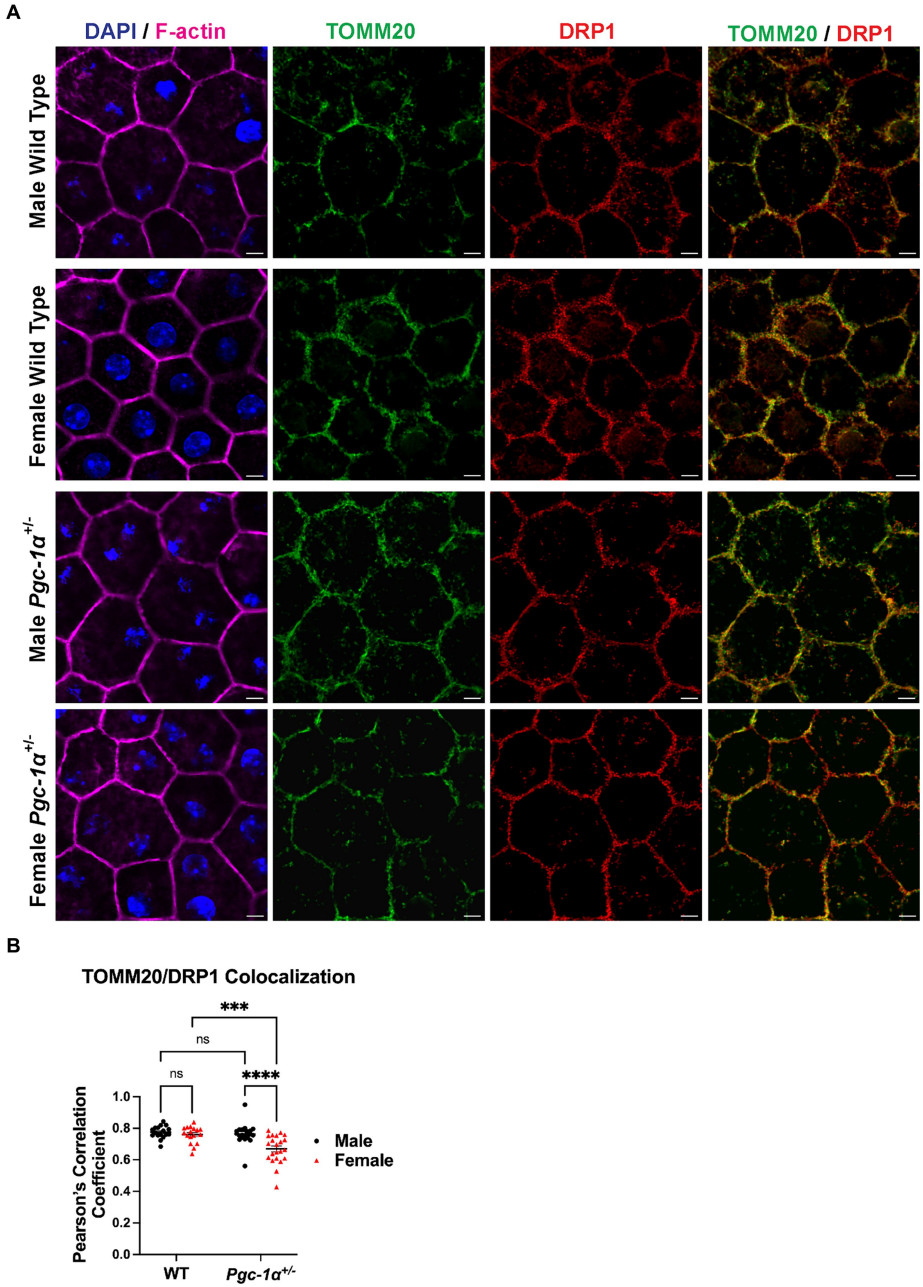
Sex-dependent regulation of mitochondrial fission in the RPE of *Pgc-1α*^+/−^ mice. **(A)** Representative confocal microscopy images of RPE flat mounts from 8mo male and female WT and *Pgc-1α*^+/−^ mice. TOMM20 (green; translocase of outer mitochondrial membrane 20), DRP1 (red; dynamin-1-like protein), F-actin (magenta), nuclei (DAPI). **(B)** Colocalization analysis of TOMM20 and DRP1 as an indicative measure of mitochondrial fission, quantified with Pearson’s correlation coefficient (Pearson’s r). *n* = 17–22 regions of interest analyzed per group; *N* = 3 animals per sex in WT, *N* = 4 animals per sex in *Pgc-1α*^+/−^. Scale bar 5 μm. Two-way ANOVA with *post hoc* Holm-Šídák test for multiple comparisons. Mean ± SEM. ^*^p ≤ 0.05, ^**^p ≤ 0.01, ^***^p ≤ 0.001, and ^****^p ≤ 0.0001.

RPE flat mounts from 8mo male and female *Pgc-1α*^+/−^ and WT mice immunolabeled with DRP1 and TOMM20 antibodies were imaged with confocal microscopy and quantitatively analyzed for colocalization Figure 5A. DRP1 is recruited onto the outer mitochondrial membrane upon activation in order to initiate mitochondrial fission (Kleele et al., 2021). The measurement of the colocalization of DRP1 and TOMM20 (as measured via Pearson’s *r*) is an indicator of mitochondrial fission events. Using RPE flat mounts from 8mo WT and *Pgc-1α*^+/−^ males and females, we found a significant interaction effect (*p* = 0.0103; two-way ANOVA factors: genotype × sex). *Post hoc* analysis revealed a significantly higher degree of colocalization between DRP1 and TOMM20 in male *Pgc-1α*^+/−^ compared to *Pgc-1α*^+/−^ females (adj. *p* < 0.0001). Moreover, *Pgc-1α*^+/−^ females had a significantly lower degree of colocalization compared to WT females (Figure 5B) (adj. *p* = 0.0002).

### Differential regulation of mitochondrial network organization

**Figure 6:**
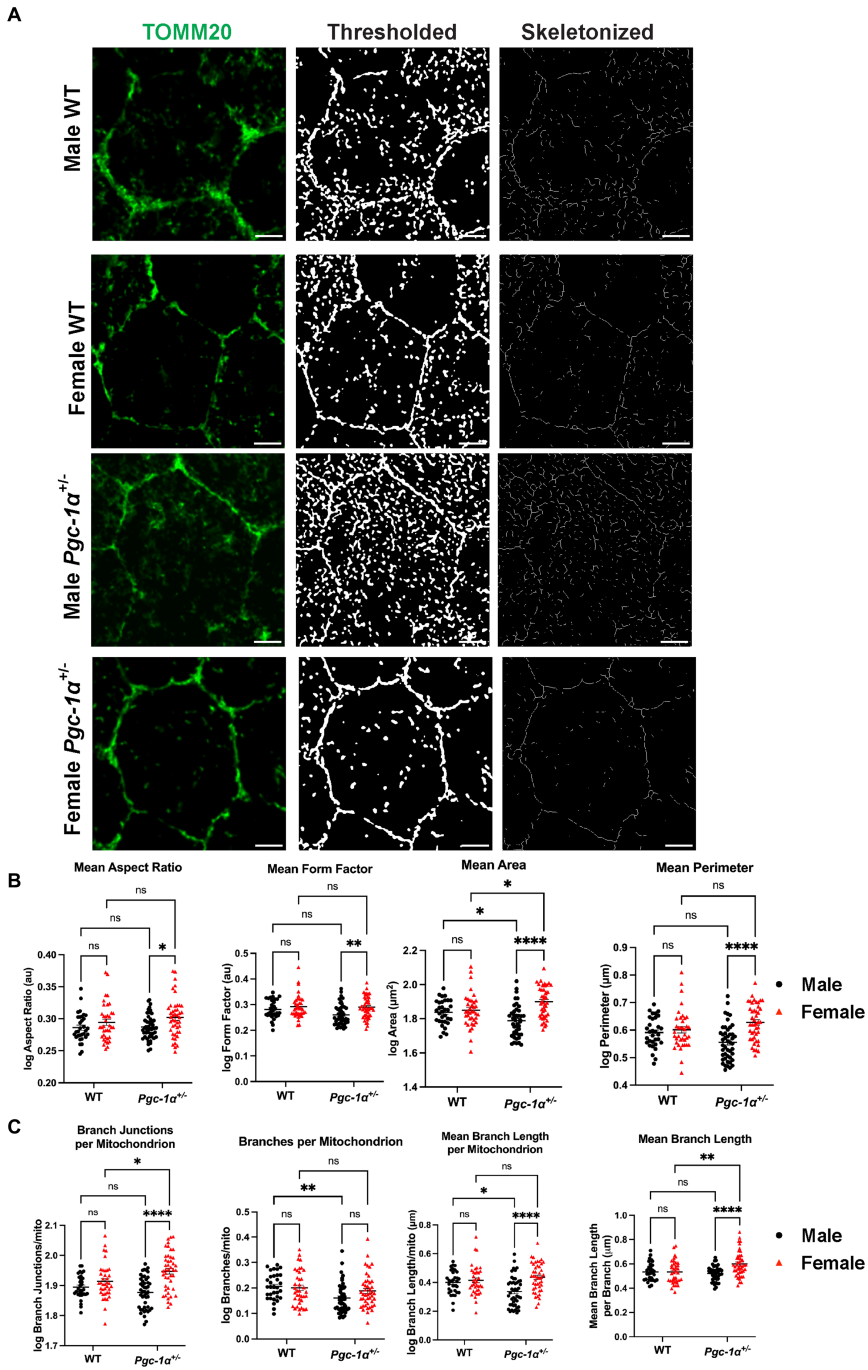
*Pgc-1α* repression differentially affects mitochondrial network organization in the RPE of males and females. Representative confocal microscopy images of mitochondrial networks in RPE flat mounts from 8mo male and female WT and *Pgc-1α*^+/−^ mice. **(A)** Mitochondria are stained with anti-TOMM20 (green). The image processing workflow using the Mitochondria Analyzer plugin is illustrated with thresholded and skeletonized versions of TOMM20-labeled mitochondria. **(B)** Statistical comparisons of mitochondrial shape descriptors mean aspect ratio, mean form factor, mean area, and mean perimeter. **(C)** Statistical comparisons of mitochondrial network connectivity and morphology parameters branch junctions per mitochondrion, branches per mitochondrion, mean branch length per mitochondrion, and mean branch length per branch. Scale bar 5 μm. Two-way ANOVA with *post hoc* Holm-Šídák test for multiple comparisons. *n* = 10–15 cells analyzed per group; *N* = 3 animals per sex in WT, *N* = 4 animals per sex in *Pgc-1α*^+/−^. Mean ± SEM. ^*^p ≤ 0.05, ^**^p ≤ 0.01, ^***^p ≤ 0.001, and ^****^p ≤ 0.0001.

To further evaluate the functional significance of the sex- and *Pgc-1α*-related differences in DRP1/TOMM20 colocalization, we carried out analyses of mitochondrial network organization and morphology in RPE flatmounts from 8mo WT and *Pgc-1α*^+/−^ mice of both sexes (Figure 6A). Using the Mitochondria Analyzer plugin on ImageJ, we assessed various measures of mitochondrial network morphology. We found a significant main effect of sex on mean mitochondrial aspect ratio (Figure 6B; *p* = 0.0047). Pairwise comparisons revealed a significantly higher mean aspect ratio in female *Pgc-1α*^+/−^ compared to male *Pgc-1α*^+/−^ (adj. *p* = 0.0228). Aspect ratio is calculated by dividing the major axis by the minor axis, and a higher aspect ratio indicates the presence of more fused mitochondria (Picard et al., 2013). Form factor is a measure of mitochondrial branching and complexity of interconnectedness (Picard et al., 2013). Lower form factor values indicate an overall morphology consisting of more round mitochondria. A significant main effect of sex was found on mean form factor (*p* = 0.0023) and was significantly lower in *Pgc-1α*^+/−^ males compared to *Pgc-1α*^+/−^ females (adj. *p* = 0.0034) (Figure 6B). Mean perimeter showed a significant interaction effect between genotype and sex (Figure 6B; *p* = 0.0030). The mean perimeter of mitochondria was significantly higher in *Pgc-1α*^+/−^ females compared to *Pgc-1α*^+/−^ males (Figure 6B; adj. *p* < 0.0001). Analysis of mean area revealed a significant interaction effect of genotype and sex (*p* = 0.0006). We observed significantly higher mean area in *Pgc-1α*^+/−^ females compared to *Pgc-1α*^+/−^ males (Figure 6B) (adj. *p* < 0.0001). Mean area of mitochondrial networks from *Pgc-1α*^+/−^ females was significantly higher compared to WT females (adj. *p* = 0.0348) and significantly higher in WT males compared to *Pgc-1α*^+/−^ males (Figure 6B) (adj. *p* = 0.0355).

Next, we analyzed per-mitochondrion (mito) descriptors of connectivity and network complexity from the thresholded and 2D skeletonized networks (Figures 6A,C). We found a significant interaction effect of genotype and sex on branch junctions per mito (*p* = 0.0044). Branch junctions per mito was found to be significantly higher in *Pgc-1α*^+/−^ females compared to *Pgc-1α*^+/−^ males (adj. *p* < 0.0001), and significantly higher in *Pgc-1α*^+/−^ females compared to WT females (Figure 6C) (adj. *p* = 0.0202). For branches per mito, we found a significant main effect of genotype (Figure 6C) (*p* = 0.0033). *Post hoc* analyses revealed a significantly lower average number of branches/mito for *Pgc-1α*^+/−^ males compared to WT males (adj. *p* = 0.0060). We observed a significant interaction effect of sex and genotype on mean branch length per mito (Figure 6C; *p* = 0.0082). Mean branch length per mito was found to be significantly higher in *Pgc-1α*^+/−^ female mice compared to *Pgc-1α*^+/−^ males (adj. *p* < 0.0001) and significantly lower in *Pgc-1α*^+/−^ males compared to WT male mice (adj. *p* = 0.0176). Mean branch length per branch in the skeletonized network was obtained via the “Analyze Skeleton” feature on Fiji (Figure 6C). Statistical analysis revealed a significant interaction effect on mean branch length (Figure 6C) (*p* = 0.0037). Mean branch length (per branch) was found to be significantly longer in female *Pgc-1α*^+/−^ compared to male *Pgc-1α*^+/−^ (adj. *p* < 0.0001). We also found a significantly longer mean branch length for female *Pgc-1α*^+/−^ as compared to female WT (adj. *p* = 0.0014).

## Discussion

### Sex- and age-dependent decline in RPE/retinal function

While aging is the main factor in RPE and retinal degeneration, including AMD, sex has also been reported to influence the prevalence of AMD. Studies have reported on a higher incidence of AMD in women compared to men across various populations (Javitt et al., 2003). However, whether sex is a risk factor in retinal degeneration remains elusive.

While there was no significant difference in c-wave ERG between WT males and females at 3mo, female WT mice showed lower RPE function compared to males at 8mo, suggesting that aging could induce a more significant reduction in RPE function in female WT mice. *Pgc-1α* repression at 3mo did not induce any reduction in RPE function in males or females compared to 3mo WT mice. A combination of aging and *Pgc-1α* repression significantly reduced RPE function in both males and females. However, female *Pgc-1α*^+/−^ - at 8mo showed lower RPE function compared to male *Pgc-1α*^+/−^.

Rod photoreceptor and inner retinal function declined in *Pgc-1α*^+/−^ mice in a sex-dependent manner. In WT mice, we did not observe sex differences in dark-adapted flash ERG a- and b-waves at either age point. This is in accordance with prior literature reporting no sex differences in ERG a- and b-waves in age-matched 2- and 12-mo C57BL/6J WT (Mazzoni et al., 2019). However, we did observe significant differences in b-wave amplitudes between 8mo *Pgc-1α*^+/−^ males and females, suggesting a sex-dependent disruption of inner retinal function in females caused by *Pgc-1α* repression. While 8mo *Pgc-1α*^+/−^ females also showed significantly reduced a- and b-wave ERG amplitudes compared to age-matched WT females, 8mo *Pgc-1α*^+/−^ and WT males showed no statistically significant differences. Importantly, no differences were observed at 3mo across any groups. Our results suggest that *Pgc-1α* inhibition affects rod photoreceptor and inner retinal function during aging in a sex-dependent manner.

The decline we observed in female *Pgc-1α*^+/−^ a- and b-waves might be complementary to the exacerbated decline in c-waves for females (Perlman, 1995). The c-wave is partly dependent on the integrity of photoreceptors, and disruptions to the complex interactions between the photoreceptors and the RPE have direct implications on c-wave amplitudes (Perlman, 1995). *Pgc-1α* might regulate RPE and retinal function and metabolic pathways through distinct mechanisms in males and females.

### Gene expression changes in antioxidant defense pathways in the RPE/retina

A recent study on the RPE-specific translatome of 6mo and 24mo C57BL/6J WT animals has reported age- and sex-specific differences in inflammation, epithelial-to-mesenchymal transition, and transepithelial transport pathways (Chucair-Elliott et al., 2024). Other studies have found sex differences in the retinal transcriptomes of C57BL/6NJ mice during aging (Du et al., 2017). Considerable sex differences in the metabolome of the RPE and neural retina during fed and fasted states in C57BL/6J mice suggest fundamental differences in the mitochondrial metabolism of males and females (Saravanan et al., 2023). The RPE of 8- to 10-week-old female C57BL/6J mice has been reported to be more susceptible to sodium iodate-induced damage and oxidative stress than males (Yang et al., 2021). Interestingly, the sodium-iodate-induced damage was also associated with a prolonged significant decrease in the expression of *Cat* in females compared to males (Yang et al., 2021). Here, we observed no differences in *Cat* expression between males and females. However, there was a significant reduction in the mRNA levels of *Cat* in female *Pgc-1α*^+/−^ compared to female WT, suggesting that *Pgc-1α* repression may significantly disrupt the expression of *Cat* in females.

We also observed a significant reduction of *Sod1* and *Sod2* expression in male *Pgc-1α*^+/−^ compared to female *Pgc-1α*^+/−^ and male WT. Conversely, *Prdx5* was upregulated in *Pgc-1α*^+/−^ mice independent of sex and even significantly higher in males than females. *Gpx1* was significantly downregulated only in male *Pgc-1α*^+/−^ compared to male WT. These observations suggest that inhibition of *Pgc-1α* differentially affects the antioxidant genes of the RPE/retina.

In humans, fundamental sex differences in antioxidant enzyme levels and antioxidant defense capacities exist throughout all stages of life, including in newborns (Diaz-Castro et al., 2016; Tiberi et al., 2023). Estrogen signaling through nuclear estrogen receptors and estradiol-induced *Nfe2l2* activity is strongly implicated in underlying sex differences in antioxidant enzymes (Tiberi et al., 2023). *Nfe2l2* was significantly reduced in *Pgc-1α*^+/−^ mice compared to WT independent of sex, consistent with our previous data on ARPE-19-*PGC1A* KO cells (Zhou et al., 2024). On the contrary, *Esr1* expression levels significantly declined in female *Pgc-1α*^+/−^ compared to female WT, indicating a regulatory role for *Pgc-1α* on *Esr1*. Liver-specific repression of *Pgc-1α* in mice has been shown to significantly decrease *Esr1* expression in female heterozygotes compared to WT controls (Besse-Patin et al., 2017). This is consistent with our results in which we observed a significant decline in *Esr1* expression in *Pgc-1α*^+/−^ compared to WT, specifically in females. It is probable that estrogen signaling in the RPE and retina is an essential factor underlying the sex differences we observed.

Our findings suggest that *Pgc-1α* repression has a differential impact on the antioxidant defenses of the RPE/retina in male and female mice. However, the mechanisms and the cellular consequences of these complex interactions in the RPE and retina remain largely unknown. This underscores the critical need for further investigation, as understanding these processes could have significant implications for the prevention and treatment of retinal diseases.

### Differential regulation of mitochondrial dynamics in males and females

Mitochondria exist not in isolation but in complex and coordinated dynamic networks that can adapt to their environment. Mitochondrial dynamics, referring to mitochondrial fission and fusion, are two essential opposing physiological processes that create broader changes in mitochondrial networks (Tilokani et al., 2018). Disruptions to the functions of the mitochondrial fission and fusion machinery underlie many progressive neurodegenerative diseases (Yapa et al., 2021). Our current understanding of mitochondrial genetics suggests that mitochondrial DNA (mtDNA) is maternally inherited (Chinnery and Hudson, 2013). Sex differences in mitochondrial dysfunctions and their implications on neuroprotection have also been discussed (Demarest and McCarthy, 2015), and mitochondria are proposed as a primary target for sex differences in diseases (Ventura-Clapier et al., 2017). However, whether mitochondria play a sex-dependent role in RPE and retinal degeneration remains to be elucidated.

To investigate whether our data on sex differences in TOMM20/DRP1 colocalization had a morphological correlation with mitochondrial networks, we analyzed the RPE mitochondrial network morphology. We did not observe any significant sex differences in WT mice, further supporting a role for *Pgc-1α* in the sex-dependent regulation of RPE mitochondrial dynamics. The analyses of mitochondria revealed significant sex- and *Pgc-1α*-related differences in mitochondrial network morphology and connectivity in RPE flat mounts. Mitochondria in the RPE of female *Pgc-1α*^+/−^ appear to favor more branching and elongation, indicative of a balance shift in mitochondrial dynamics to favor fusion. Conversely, the RPE of *Pgc-1α*^+/−^ males appear to have less branching and form fewer complex networks. Thus, males seem to favor mitochondrial fission in response to the *Pgc-1α* repression. Mitochondrial heterogeneity within RPE cells is an emerging field that is receiving increasing amounts of attention (Rzhanova et al., 2024; Tan et al., 2022). While performing our imaging, we took into account the heterogeneity across RPE cells by analyzing multiple areas from each flat mount.

Collectively, our study reveals that *Pgc-1α* repression induces a sex-dependent decline in RPE and retinal function during aging in mice. The differences in RPE and retinal function might be partly due to the differential regulation of mitochondrial homeostasis by *Pgc-1α* in males and females. Further investigations into mitochondrial dysfunction in the RPE during aging open new avenues for a sex-dependent therapeutic approach in age-related RPE and retinal degeneration.

## Data Availability

The original contributions presented in the study are included in the article/supplementary material, further inquiries can be directed to the corresponding author/s.

## Supplementary material

Supplementary Table 1 Primer sequences used in qPCR experiments.

Supplementary Table 2 ANOVA tables for Figure 1.

Supplementary Table 3 ANOVA tables for Figure 2.

Supplementary Table 4 ANOVA tables for Figure 3.

Supplementary Table 5 ANOVA tables for Figure 4.

Supplementary Table 6 ANOVA tables for Figure 5.

Supplementary Table 7 ANOVA tables for Figure 6.
